# Bringing Executive Function Testing Online: Assessment Validation Study

**DOI:** 10.2196/75687

**Published:** 2025-07-14

**Authors:** Mouna Attarha, Ana Carolina de Figueiredo Pelegrino, Lydia Ouellet, Sarah-Jane Grant, Etienne de Villers-Sidani, Thomas Van Vleet

**Affiliations:** 1 Posit Science Corporation, 160 Pine St Suite 200, San Francisco, CA, 94111, United States, 1 415-394-3100 ext 505; 2 Montreal Neurological Institute and Hospital, McGill University, Montreal, QC, Canada

**Keywords:** executive function, web-based cognitive assessment, brain health, cognitive status, National Institutes of Health Executive Abilities: Measures and Instruments for Neurobehavioral Evaluation and Research, NIH EXAMINER, assessment accessibility

## Abstract

**Background:**

Executive function encompasses a set of higher-order cognitive processes, including planning, cognitive flexibility, and inhibitory control, that are essential for goal-directed behavior. These abilities are adversely affected by age, with executive dysfunction ultimately impairing the performance of activities of daily living.

**Objective:**

This study aimed to assess the validity of a computerized cognitive assessment in predicting executive function performance in healthy older adults.

**Methods:**

This retrospective analysis utilized baseline data from the Improving Neurological Health in Aging via Neuroplasticity-Based Computerized Exercise (INHANCE) trial. The study provides normative data for cognitively healthy older adults (aged 65 years and above) and evaluates the usability and validity of Freeze Frame, a cognitive assessment available on the BrainHQ platform. Performance on Freeze Frame was analyzed in relation to self-reported demographic variables and neuropsychological function, using a standardized measure of executive function, the National Institutes of Health Executive Abilities: Measures and Instruments for Neurobehavioral Evaluation and Research (NIH EXAMINER).

**Results:**

The intent-to-treat analysis included 92 cognitively healthy older adults (mean age 71.9, SD 4.86, range: 65‐83 years), of whom 66% (61/92) were female, with a mean education level of 16.45 (SD 3.40, range: 9‐27) years. Performance on Freeze Frame was modestly associated with executive function scores on NIH EXAMINER (*P*=.02), accounting for 6.8% of the variance. The assessment showed a small but statistically significant relationship to age (ρ=−0.22, *P*=.046) and gender, with no significant influence of education. Psychometric evaluation supported its usability, with an average completion time of 4 (SD 0.16) minutes.

**Conclusions:**

Freeze Frame is a brief, scalable, and accessible computerized cognitive assessment with demonstrated concurrent validity for executive function. Its efficiency and ease of administration across internet-connected devices suggests potential applications for cognitive screening. Future research should explore its utility in detecting executive dysfunction in clinical populations and its potential role in predicting functional performance across activities of daily living.

## Introduction

Executive function is a critical domain of cognition that influences functional independence [1-5]. In healthy older adults, executive function abilities are closely associated with essential activities such as self-care, financial management, transportation use, and meal preparation. These skills support the capacity to make decisions, control action, and adapt to novel situations, which are necessary for daily living. However, executive function is known to decline with age [6,7]. Age-related changes can contribute to difficulties in maintaining autonomy and increase reliance on caregivers and support systems. Assessing executive function decline in aging populations facilitates the early identification of individuals at risk for functional impairments.

The National Institutes of Health Executive Abilities: Measures and Instruments for Neurobehavioral Evaluation and Research (NIH EXAMINER) was developed to provide a comprehensive, standardized assessment of executive function [8]. Traditional in-person neuropsychological tests are often limited in their ability to capture the complex, multifaceted nature of executive function. Computerized assessment batteries such as Cogstate, Cognifit, CNS Vital Signs, CANTAB, and NIH Toolbox are broader-purpose neurocognitive batteries that largely aim to evaluate general cognition by including domains such as speed, attention, memory, language, and emotion processing, with executive functioning being relatively underrepresented within the overall battery. NIH EXAMINER was designed to address these gaps by incorporating multiple tasks to reliably and validly evaluate various aspects of executive function [9]. Research utilizing NIH EXAMINER has shown strong associations between executive function and age, due to changes in prefrontal cortex function [10]. Additionally, studies have widely used NIH EXAMINER in clinical research, showing sensitivity to executive dysfunction across neurodevelopmental conditions such as attention deficit disorder, acquired conditions such as traumatic brain injury, and neurodegenerative diseases such as Alzheimer disease and related dementias [11]. Performance deficits on NIH EXAMINER have been associated with impairments in activities of daily living [12]. As a result, NIH EXAMINER serves as a valuable tool for both research and clinical applications, aiding in the detection of executive dysfunction and informing targeted interventions to support cognition and functional independence.

There remains a growing need for scalable, computerized cognitive assessments due to significant barriers of traditional methods, such as prolonged administration times, clinician training, and the burden of patient travel. Addressing these limitations, this study examines the usability and preliminary validity of the Freeze Frame assessment to evaluate inhibitory control in a cohort of healthy older adults (aged 65 years and above) from the Improving Neurological Health in Aging via Neuroplasticity-Based Computerized Exercise (INHANCE) trial [13]. Inhibitory control represents a critical subprocess of executive functioning, involving the rapid suppression of prepotent or automatic responses in favor of task-relevant actions to regulate behavior in dynamic environments and support cognitive flexibility, working memory, and goal maintenance [14,15]. Empirical evidence from neuroimaging and meta-analyses have demonstrated that impairments to inhibitory control are observed across neurodevelopmental, psychiatric, and neurodegenerative diseases [16-19]. Inhibitory control therefore serves as a sensitive and mechanistically informative index of executive function integrity, making it a valuable target for cognitive assessment. Virtually all existing batteries of general cognition (eg, CANTAB, Cognifit, CNS Vital Signs) include at least 1 measure of inhibitory control.

Freeze Frame is similar to other measures of inhibitory control, particularly the Sustained Attention to Response Task (SART) [20]. Response contingencies in Freeze Frame were inspired by SART and work completed in hemispatial neglect and traumatic brain injury [20]. However, the timing varies considerably between the 2 tasks in that Freeze Frame utilizes a variable intertrial interval whereas SART has a consistent intertrial interval. The advantage of a variable time interval is that unlike consistent intertrial intervals, which increases attentional lapses and response anticipations [21], the unpredictable timing of the appearance of each image in Freeze Frame requires a more alert state and enhanced response control [22]. The most unique aspect of Freeze Frame apart from the SART or Conners Continuous Performance Task [23] is the real-time adaptation of the core features of the assessment, including target frequency, the timing of the intertrial interval, and the variable and often longer duration of the task that allows time for participants to wane in their sustained attentional and inhibitory control capacities. These novel task parameters distinguish Freeze Frame from other measures of inhibitory control.

Although computerized batteries of global cognition are preferred when a comprehensive evaluation of neurocognitive profiles is needed to assist with diagnostic decisions, a single brief inhibitory control task offers several advantages in terms of speed, scale, and frequent monitoring. A targeted measure can provide interpretable performance metrics without confounding from broader executive or global cognition domains. Short completion times make brief assessments well-suited for clinical use where patient burden must be minimized, longitudinal studies that require daily or weekly monitoring over time, or high-throughput research that requires remote testing of hundreds, thousands, or millions of participants. Finally, assessments that do not require clinical training or specialized hardware can be deployed by patients using mobile devices at home and monitored by clinicians or researchers remotely.

This study evaluates the psychometric properties of Freeze Frame, its associations with demographic variables (ie, age, gender, and education), and its concurrent validity relative to NIH EXAMINER. The findings provide preliminary data that contribute to the growing need for scalable, digital tools that assess executive function, offering a promising avenue for identifying individual differences in cognition and advancing research in aging.

## Methods

### Setting, Design, and Sample

Participants were recruited near McGill University, Canada. Participants were community-dwelling, healthy older adults aged 65 years and above. The data reported reflect the intent-to-treat population (N=92) from the INHANCE randomized clinical trial (see published protocol for details [13]). The study enrolled participants from July 2021 to December 2023, with the final follow-up in June 2024.

### Participants and Inclusion and Exclusion Criteria

Participants were community-dwelling, healthy older adults. Inclusion criteria included individuals aged 65 years or older, proficient in English or French, capable of fulfilling study requirements, and cognitively intact with a Montreal Cognitive Assessment total score of ≥23, a cut-off that optimizes diagnostic sensitivity and specificity [24]. Exclusion criteria included neurocognitive disorders, suicidal ideation, major depression indicated by a total score of >10 on the Geriatric Depression Scale–Short Form, prior experience using BrainHQ within the past 5 years, concurrent clinical trial participation, pregnancy, substance abuse, or medical conditions hindering study engagement. Full details are provided in the protocol [13].

### Freeze Frame Assessment

**Figure 1.**
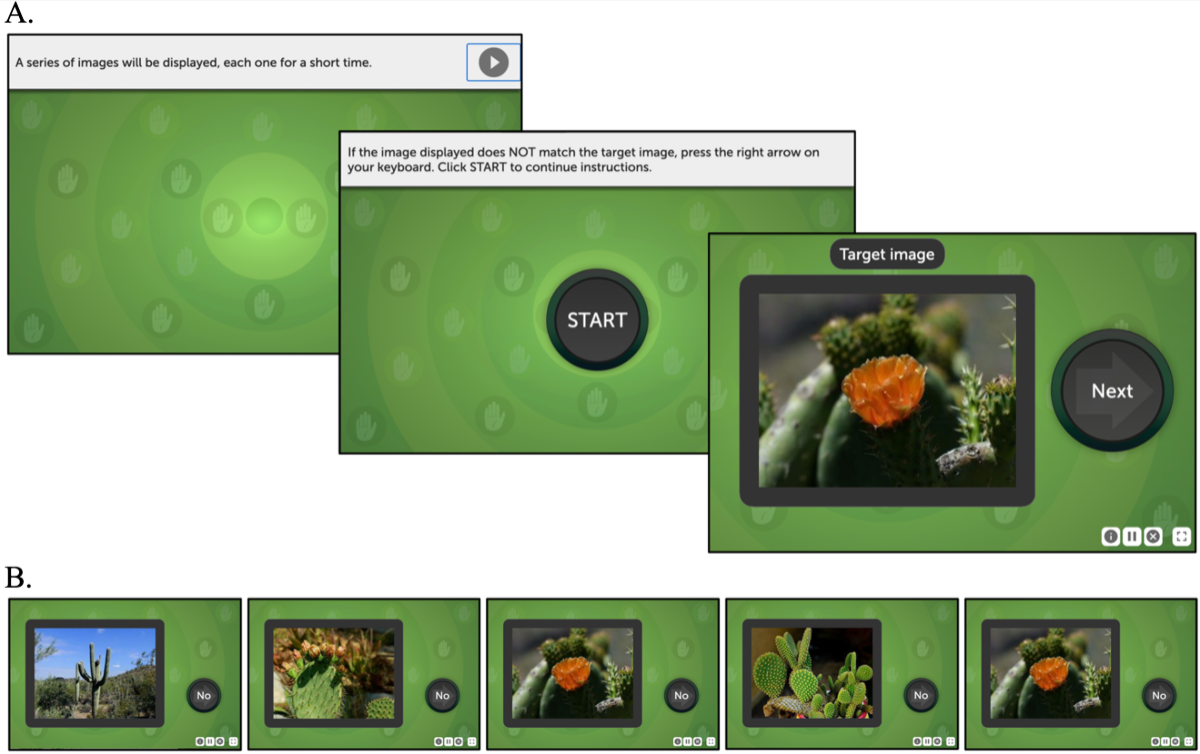
The Freeze Frame computerized cognitive assessment. Participants remember a target image presented at the start of the block after which a rapid stream of targets and foils are interleaved with unequal probability. Participants withhold a motor response to all targets and enter a speeded response to all foils. (**A**) Guided tutorial presenting the target image. (**B**) Example of sequentially presented trials of the target and sample foils. Responses are correct if the participant selects “no” to all foil images within a limited response window.

The Freeze Frame assessment is a cognitive task designed to measure speeded inhibitory control using a reverse go/no-go paradigm [25-27]. Participants are shown a target image at the beginning of each block, followed by a rapid sequence of images consisting of both targets and foils, presented with unequal probabilities under constrained viewing durations. The interstimulus interval varied randomly between 500 and 1500 milliseconds. Participants were instructed to withhold their response when a target appeared while executing a speeded motor response to all foil stimuli. See Figure 1 for a visual representation of the task structure.

The adaptive dimension of the assessment was target frequency, with lower target frequencies increasing task difficulty. There are 7 possible target frequency levels: 40%, 35%, 30%, 25%, 20%, 15%, and 10%. All participants began the assessment at a target frequency of 30%. Adaptation occurred over 5 epochs, with each epoch consisting of 30 trials. Performance-based adjustments to target frequency were made based on the abilities of each participant. If the participant correctly withheld responses to at least 80% of targets and correctly responded to at least 80% of foils, the task progressed to a more difficult level (ie, a lower target frequency). If these accuracy thresholds were not met, the task became easier by increasing the target frequency on the subsequent epoch.

The raw threshold score was recorded as an integer ranging from 1 to 7, where 1 corresponded to the easiest condition (40% target frequency) and 7 represented the most challenging condition (10% target frequency). While threshold scores were used in the raw data output, these thresholds were converted to mean accuracy, as discussed in the protocol [13].

### Ethical Considerations

The study was developed in accordance with the Declaration of Helsinki guidelines and was approved by the Western Institutional Review Board (IRB00000533) and Research Ethics Board of McGill University Health Centre (2020‐6474). All participants provided written informed consent.

Study data were recorded into a secure, web-based electronic case report form at the study site through the Longitudinal Online Research and Imaging System (LORIS). This system meets relevant privacy and security standards for electronic trial data entry and storage, as well as the Health Insurance Portability and Accountability Act and Personal Information Protection and Electronic Documents Act standards for confidentiality and privacy [28]. Following consent, each participant was assigned a standardized Participant Identification Number composed of digits to identify the study and digits to identify the participant. All electronic case report form data entries were deidentified, using the Participant Identification Number and not the participant’s name.

Participants received CAD $40 (approximately US $30) for completing the baseline visit, which included the measures described below. Payment followed the completion of the visit. Additional details are provided in the protocol [13].

The study was registered on November 4, 2019, before participant enrollment under the name “Improving Neurological Health in Aging Via Neuroplasticity-based Computerized Exercise (INHANCE)” with identification number NCT04149457.

### Measures

Blinded staff conducted assessment administration and scoring. Following informed consent, participants completed a structured clinical interview, the Freeze Frame assessment, and a validated neuropsychological assessment of executive function (NIH EXAMINER). The structured clinical interview (20 minutes, in person) collected key demographic (eg, age, gender, education). The Freeze Frame assessment (8 minutes, in person) evaluated inhibitory control. NIH EXAMINER (20 minutes, in person) assessed executive function [8], and performance was measured using the executive composite score [10], which is automatically generated by the assessment program and converted to a *z* score per the registered protocol [13].

### Analyses

#### Overview

Recruitment spanned from July 2021 to December 2023. The final follow-up visit was on June 7, 2024, and the study team was unblinded on June 14, 2024, after database lock. For all analyses, a *P*<.05 determined statistical significance [13].

#### Demographic Characteristics

Participants self-reported their age, gender, and educational level and completed the NIH EXAMINER assessment. Descriptive statistics including arithmetic means, SDs, and ranges were provided for continuous variables (age, education, NIH EXAMINER) to summarize central tendency and variability. For categorical variables such as gender, we calculated the frequency and percentage of participants who selected each response option.

#### Assessment Characteristics

To assess the usability of the Freeze Frame assessment, we used descriptive statistics (arithmetic mean and SD), characteristics of the distribution (skew and kurtosis), and psychometric properties including the performance histogram (the proportion of participants achieving each possible assessment score), as well as the time spent completing the assessment. To assess the presence and extent of ceiling and floor effects, we determined the proportion of participants who obtained the lowest and highest possible scores. A high frequency of extreme scores may suggest limitations in the assessment’s ability to discriminate performance across different levels of ability.

#### Associations Between Freeze Frame, Demographics, and NIH EXAMINER

To examine the relationship between performance on the Freeze Frame assessment and key demographic variables, we used Spearman ρ for age and education. For gender-based comparisons, we applied the Wilcoxon rank-sum test to evaluate differences in assessment performance between women and men. We used Pearson *r* to investigate the relationship between assessment performance and NIH EXAMINER. We further used simple linear regression to evaluate the relationship between Freeze Frame and NIH EXAMINER to assess the extent to which performance on the assessment could account for variance in executive function scores, thereby providing concurrent validity with an established neuropsychological measure.

## Results

### Demographic Characteristics

Participants (N=92) had a mean age of 71.90 (SD 4.86; range: 65‐83) years and a mean education level of 16.45 (SD 3.40, range: 9‐27) years. Of the 92 participants, 61 (66%) identified as female and 31 (34%) identified as male. The baseline NIH EXAMINER executive composite mean was 0.43 (SD 0.57; range: −1.50 to 1.52; see Table 1). A complete characterization of the intent-to-treat population has been published previously [29].

**Table 1.** Baseline characteristics of the intent-to-treat population (N=92).

Characteristic	Participants
Age (years), mean (SD)	71.9 (4.9)
Education (years), mean (SD)	16 (3.4)
Gender, n (%)	
Female	61 (66)
Male	31 (34)
Race, n (%)	
White	88 (96)
Asian	2 (2)
Black or African American	1 (1)
American Indian or Alaska Native	0 (0)
Native Hawaiian or other Pacific Islander	0 (0)
More than one race	0 (0)
Unknown or not reported	1 (1)
Ethnicity, n (%)	
Not Hispanic or Latino	91 (99)
Hispanic or Latino	0 (0)
Unknown or not reported	1 (1)
Freeze Frame (accuracy), raw score (SD)	92.01 (4.70)
NIH EXAMINER^a^, executive composite score (SD**)**	0.43 (0.57)

a NIH EXAMINER: National Institutes of Health Executive Abilities: Measures and Instruments for Neurobehavioral Evaluation and Research.

### Assessment Characteristics

**Figure 2.**
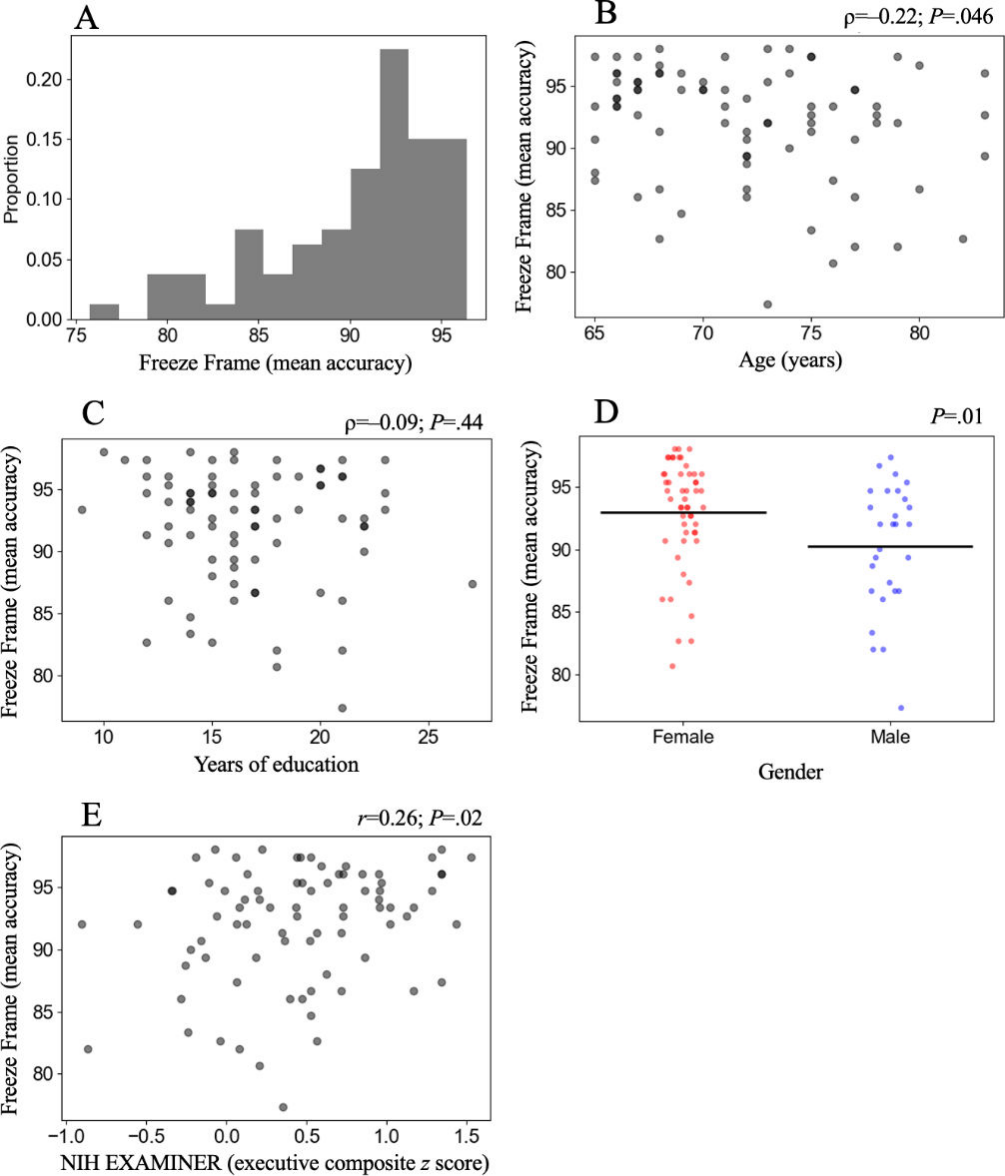
Outcome measures. (**A**) Performance histogram of Freeze Frame scores (mean accuracy) across the intent-to-treat population. Association of Freeze Frame with demographic characteristics including (**B**) age, (**C**) years of education, and (**D**) gender. (**E**) Association of Freeze Frame with the NIH EXAMINER executive composite, a standard validated measure of executive function. NIH EXAMINER: National Institutes of Health Executive Abilities: Measures and Instruments for Neurobehavioral Evaluation and Research.

The distribution of Freeze Frame assessment scores is displayed in Figure 2A. Mean accuracy was 92.01% (SD 4.70%), with a skew of −0.99 and a kurtosis of 0.37. With respect to extreme scores, the highest recorded mean accuracy was 98%, achieved by 3 (3.75%) participants with no participant obtaining a perfect score. The lowest recorded mean accuracy was 77.33%, observed in 1 (1.25%) participant. Participants required an average of 4.27 (SD 0.16) minutes to complete the assessment.

### Associations Between Freeze Frame, Demographics and NIH EXAMINER

Performance on the Freeze Frame assessment showed a small but statistically significant negative association with age (ρ=−0.22, *P*=.046), and a significant effect of gender with women performing better than men (*P*=.01). In contrast, the association between Freeze Frame performance and years of education was nonsignificant (ρ=−0.09, *P*=.44; see Figure 2B–D).

With respect to executive function, Freeze Frame performance was positively correlated with the NIH EXAMINER executive composite score (*r*=0.26; *P*=.02; see Figure 2E). Linear regression showed that Freeze Frame scores were significantly associated with the NIH EXAMINER executive composite score (*F*_1,78_=5.693; *P*=.02) and modestly accounted for 6.8% of the variance (*R*²=0.068).

### Missing Data

A total of 12 participants (13% of the sample) did not have mean accuracy scores that could be calculated from threshold scores due to administrative errors. Missing data were not imputed for baseline data of predefined exploratory outcomes data, and Freeze Frame associations reflect participants from the intent-to-treat population with complete data at baseline. There were no statistical differences between those with versus without Freeze Frame mean accuracy scores across demographic variables or NIH EXAMINER scores.

## Discussion

### Principal Findings

Freeze Frame assesses inhibitory control and, in this study, captured modest variance in overall executive function, with better Freeze Frame performance associated with better NIH EXAMINER performance. Older participants exhibited lower accuracy on the task compared to participants who were relatively younger. Educational background did not influence task performance. Extreme scores were minimal, suggesting that the assessment did not have notable ceiling or floor effects. The duration of the assessment was brief, at 4 minutes, and the SD suggests that completion times were stable and did not introduce significant variability due to differences in participant pacing. These results provide preliminary evidence that Freeze Frame may serve as a useful indicator of executive function and that healthy older adults are generally able to complete the assessment within a feasible administration time.

Performance on most cognitive assessments declines with age, largely due to the complex interplay of structural, functional, and neurochemical changes that occur in the aging brain [30]. While considerable individual differences exist, age-related reductions in cortical thickness, functional connectivity, and neuromodulatory control generally result in measurable decreases in cognitive function over one’s lifespan, such as slower information processing and diminished executive functioning [7]. In contrast, assessments associated with crystallized intelligence, such as general content knowledge, vocabulary, and spelling, often remain stable or improve with age [31]. In this study, performance on the Freeze Frame assessment was found to decrease modestly with age, with relatively younger participants outperforming older participants. This pattern is consistent with the intended design of Freeze Frame to evaluate cognitive networks that are vulnerable to age-related decline [25-27,32,33].

In this study, women demonstrated significantly better performance than men on the Freeze Frame assessment, which is consistent with previous evaluations of Freeze Frame [34] as well as other validated assessments of executive function [35]. This finding aligns with prior research suggesting that women often exhibit better response inhibition and cognitive control, particularly in tasks requiring rapid decision-making using go/no-go paradigms [36]. These differences may be due to strategy differences [37] or may be influenced by sociocultural factors, including differences in early-life experiences and cognitive engagement. Neurobiological factors, including structural and functional variations in prefrontal networks, which play a critical role in executive function abilities, may also contribute to these observed performance disparities [38]. The findings underscore the importance of considering sex-based differences in cognitive assessments and highlight the need for further research to explore underlying mechanisms driving such differences and their implications for cognitive aging and disease vulnerability.

The relatively small proportion of variance in NIH EXAMINER scores explained by Freeze Frame (6.8%) suggests that the assessment captures limited aspects of the broader executive function construct. The modest shared variance highlights that most individual differences in executive function remain unexplained by Freeze Frame alone. This is not unexpected given that the NIH EXAMINER executive composite score integrates performance across multiple domains of executive function, whereas Freeze Frame is narrowly focused on a speeded version of inhibitory control. Freeze Frame may nevertheless serve as a useful, fully remote, and self-administered proxy within research or clinical screening contexts that rely on rapid evaluation. The findings reported further highlight the value of incorporating additional tasks using the same remote assessment methodology (such as those capturing other aspects of executive function like working memory) to develop a comprehensive evaluation of overall functioning.

### Limitations

The generalizability of the study findings is constrained by the limited demographic diversity of the participant sample, which may not fully represent racial and ethnic minority groups [29]. As a result, the applicability of these findings to more diverse and lower-education populations remains uncertain, beyond preliminary evidence from a prior study involving a sample with greater racial and educational diversity [34], and warrants further investigation. Additionally, the age range of participants (65-83 years) was restricted due to the study’s inclusion criteria, potentially limiting the ability to detect associations across a broader age spectrum. Another methodological limitation pertains to the Freeze Frame assessment specifically, where 13% of threshold scores could not be converted to mean accuracy, which reduced the analytic sample to 80 and likely diminished the statistical power of the analyses and may have attenuated the magnitude of observed effects.

### Strengths

The INHANCE study is distinguished by several methodological strengths, including a rigorous study design, the implementation of stringent inclusion criteria, and adherence to a prespecified statistical analysis plan. A key advantage of this study is the use of a computerized assessment platform, which enhances standardization, reliability, and efficiency in data collection. Furthermore, the digital nature of the assessment allows for broad scalability, making it accessible to individuals across diverse geographic and socioeconomic backgrounds who have access to an internet-enabled device. This broad accessibility supports the potential for large-scale implementation in both research and clinical settings pending further validation studies.

### Conclusions

Findings from this study highlight the preliminary sensitivity of Freeze Frame to evaluate higher-order cognitive abilities. Freeze Frame may serve as a scalable behavioral proxy for detecting executive function deficits in aging populations. Future research should investigate whether lower performance on this task is an early indicator of heightened risk for cognitive decline. Additionally, further studies are needed to determine the utility of Freeze Frame in clinical populations known to exhibit executive dysfunction, such as individuals with attention deficit disorder, traumatic brain injury, or mild cognitive impairment. Examining the predictive validity of Freeze Frame in relation to real-world functional decline will be critical in establishing its clinical applicability as a screening tool.

## Abbreviations

INHANCE: Improving Neurological Health in Aging via Neuroplasticity-Based Computerized Exercise
LORIS: Longitudinal Online Research and Imaging System
NIH EXAMINER: National Institutes of Health Executive Abilities: Measures and Instruments for Neurobehavioral Evaluation and Research
SART: Sustained Attention to Response Task
